# Immunomodulatory and cytotoxic effects of dental methacrylates: a narrative review focusing on 2-hydroxyethyl methacrylate and triethylene glycol dimethacrylate

**DOI:** 10.3389/fbioe.2026.1850156

**Published:** 2026-06-16

**Authors:** Sara Alizadehgharib, Gilda Otterlind, Ulf Örtengren

**Affiliations:** 1 Department of Oral Microbiology and Immunology, Institute of Odontology, Sahlgrenska Academy, University of Gothenburg, Gothenburg, Sweden; 2 Department of Cariology, Institute of Odontology, Sahlgrenska Academy, University of Gothenburg, Gothenburg, Sweden; 3 Department of Materials Science and Applied Mathematics, Faculty of Odontology, Malmö University, Malmö, Sweden

**Keywords:** antibodies, biocompatibility, cytokines, cytotoxicity, immune system, methacrylates

## Abstract

Polymer resin-based dental materials may release unreacted monomers, including 2-hydroxyethyl methacrylate (HEMA) and triethylene glycol dimethacrylate (TEGDMA), which can interact with host tissues and influence biological responses. This narrative review summarizes current evidence on the immunomodulatory and cytotoxic effects of these commonly used dental methacrylates. A total of 42 *in vitro* and *in vivo* studies were included. The available evidence indicates that HEMA and TEGDMA can modulate immune responses, alter cytokine production, induce oxidative stress and DNA damage, and reduce cell viability, with TEGDMA generally appearing more cytotoxic than HEMA. Both monomers have been reported to increase pro-inflammatory cytokine release and to exhibit adjuvant-like effects in the presence of antigens. In contrast, co-exposure to bacterial components such as lipopolysaccharide and lipoteichoic acid was associated with attenuated microbe-induced cytokine responses, indicating a context-dependent immunomodulatory profile. Overall, the biological effects of HEMA and TEGDMA vary according to concentration, cell type, and experimental conditions, with both immunostimulatory and immunosuppressive effects reported. These findings highlight the complex biological activity of dental methacrylates and underscore the need for careful evaluation of their biocompatibility and clinical safety.

## 1 Introduction

**FIGURE 1:**
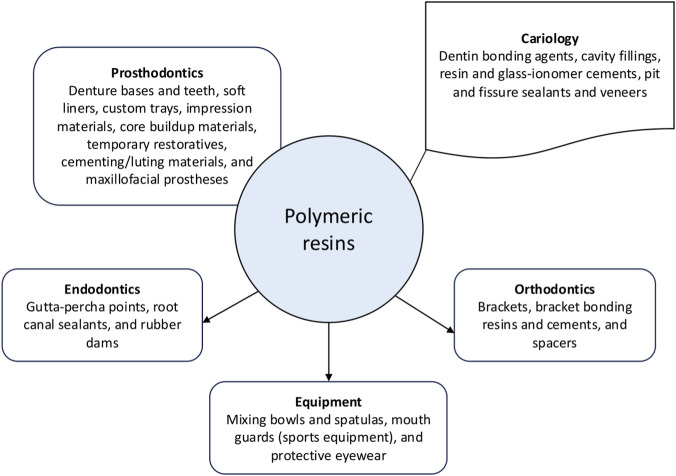
Overview of polymer resin-based materials used in clinical practice. The figure illustrates the wide range of clinical use across dental specialties, including prosthodontics, cariology, orthodontics, endodontics, and others.

Polymer resins, consisting of monomers combined with other components, are extensively used in dentistry. They play a central role across multiple disciplines, including prosthodontics, cariology, orthodontics, endodontics, and dental equipment, as summarized in Figure 1. Over recent decades, these materials have largely replaced traditional restorative materials such as dental amalgam. Following Sweden’s phase-out of amalgam in 1994 and its subsequent environmental prohibition in 2009 (Government of Sweden, 2002; Brodén, 2009), methacrylate-based composites, adhesives, cements, and glass ionomers became the predominant materials in modern dentistry (Aalto-Korte et al., 2007). Despite their widespread use, polymer-based restoratives may release components with biological effects, raising concerns regarding their biocompatibility (Björkman et al., 2017; Stanislawski et al., 2003; Schweikl and Schmalz, 1999).

### 1.1 Dental methacrylates

**FIGURE 2:**
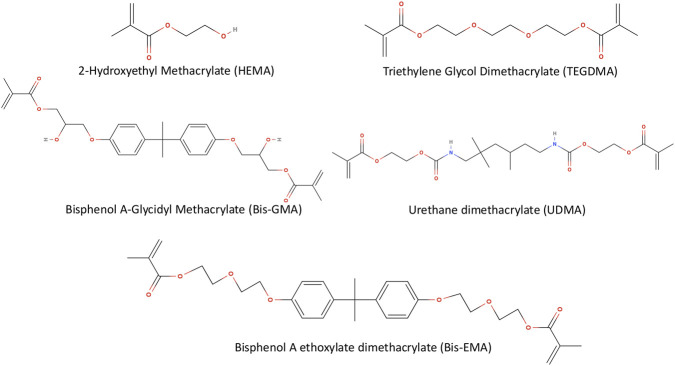
Chemical Structures of Common Dental Methacrylate Monomers. The figure illustrates the structures of methacrylates used in dental materials. Depicted are 2-Hydroxyethyl methacrylate (HEMA), Triethylene glycol dimethacrylate (TEGDMA), Bisphenol A-glycidyl methacrylate (bis-GMA), Urethane dimethacrylate (UDMA), and Bisphenol A ethoxylate dimethacrylate (bis-EMA). Chemical structures of HEMA, TEGDMA, Bis-GMA, UDMA, and bis-EMA were generated using MolView (MolView.org) with structural data retrieved from PubChem.

Dental methacrylates are composed of various methacrylic acid esters and form the matrix of resin-based composites (RBCs) and adhesives. RBCs contain a continuous monomer phase and a discontinuous filler phase bound by silane coupling agents (Zimmerli et al., 2010; Fleming, 2014; Cornelio et al., 2014). Most RBCs are composed of several types of methacrylates such as bisphenol A-diglycidyl methacrylate (bis-GMA), ethoxylated bisphenol-A dimethacrylate (bis-EMA), urethane dimethacrylate (UDMA), and triethylene glycol dimethacrylate (TEGDMA) (Fleming, 2014; Cornelio et al., 2014; Peutzfeldt, 1997). RBCs are bonded to tooth structures using multi-component adhesive systems containing functional methacrylate monomers. Hydroxyethyl methacrylate (HEMA) is commonly present in adhesive formulations due to its hydrophilic properties (Van Landuyt et al., 2007), and is also found in resin-modified glass ionomers (Palmer et al., 1999). The chemical structures of five important methacrylates are found in Figure 2.

Polymerization, initiated by the creation of free radicals via chemical, light, heat, or microwave energy, links monomers via covalent bonds to a three-dimensional polymer network (Peutzfeldt, 1997; Kostić et al., 2022). Most methacrylates are di- or multifunctional and viscosity increases during curing. Because of gelation, the mobility of reactive methacrylate groups becomes restricted during curing, limiting the extent of monomer conversion and leaving some double bonds unreacted. As a consequence, some double bonds will remain unreacted. Light-cured RBCs typically reach 50%–65% conversion (Peutzfeldt, 1997; Rueggeberg and Craig, 1988; Ferracane and Greener, 1986), increasing slightly post-cure (Johnston et al., 1985; Schneider et al., 2006). The actual residual monomer content is considered low (∼3%) (Ferracane, 1994), with about 90% of leachable monomers released within 24 h (Ferracane, 1994; Schmalz et al., 2011). Inadequate curing or handling, as well as intra-oral mechanical, hydrolytic, and enzymatic degradation, can further enhance monomer release and exposure risks for patients and dental personnel (Ekstrand et al., 1998; Wallenhammar et al., 2000; Schedle et al., 2007; Wrangsjö et al., 2001; Van Landuyt et al., 2011; Schmalz and Arenholt-Bindslev, 2009; Peutzfeldt and Asmussen, 1992).

Released monomers can enter the body via oral mucosa, skin absorption, inhalation of volatiles (Hagberg et al., 2005; Piirila et al., 1998), ingestion (Kwon et al., 2015), or diffusion to the pulp through dentinal tubules (Hörsted-Bindslev et al., 2003; Noda et al., 2002). Ingestion and pulpal diffusion mainly affect patients, whereas dermal and inhalational exposure pose greater risks for dental personnel (Wallenhammar et al., 2000; Van Landuyt et al., 2011; Nilsen et al., 2019). Consequently, these monomers have raised concerns regarding potential toxicity (Kostić et al., 2022) and allergic reactions (Wallenhammar et al., 2000; Wrangsjö et al., 2001; Kiec-Świerczyńska, 1996). Due to the handling of uncured materials, dental personnel are at risk of respiratory distress or hand dermatitis from methacrylate exposure (Aalto-Korte et al., 2007; Kostić et al., 2022; Syed et al., 2015; Lindström et al., 2002). Allergic contact dermatitis, characterized by dryness, cracking, itching, and swelling (Kostić et al., 2022; Wallenhammar et al., 2000; Schedle et al., 2007), results from repeated hapten-mediated T-cell sensitization (Vocanson et al., 2009). Monomers such as TEGDMA, HEMA, Ethyl methacrylate (EMA), and Diethylene glycol diacrylate (DEGDA) have been associated with these effects (Aalto-Korte et al., 2007; Wallenhammar et al., 2000; Aalto-Korte et al., 2010), and cross-allergies between methacrylates may occur (Rusteme et al., 1998). In patients, adverse reactions can manifest as lichenoid lesions, stomatitis, or ulcers with pain and burning (Björkman et al., 2017; Schedle et al., 2007; Schmalz et al., 2016). Methacrylates can disrupt cellular mechanisms underlying these reactions, though clinically relevant molecular pathways remain insufficiently understood. Among the various dental methacrylates, low–molecular-weight monomers such as HEMA and TEGDMA are of particular interest due to their higher mobility, release potential, and biological reactivity.

### 1.2 Immune system

The immune system comprises the innate and adaptive branches, which interact bidirectionally during immune responses (Parkin and Cohen, 2001). The innate system provides immediate defense through neutrophils, macrophages, NK cells, granulocytes, and acute-phase proteins (Dahlén et al., 2012), while the adaptive system uses antigen-specific T and B cells to generate antibodies (IgG, IgM, IgA, IgE, IgD) and cytokines that regulate inflammation (Parkin and Cohen, 2001; Kany et al., 2019). Since dental methacrylates can interact with host tissues, they have the capability to influence these immune pathways. In some individuals, this can trigger hypersensitivity, i.e., reproducible immune reactions to otherwise harmless stimuli (Johansson et al., 2006). Released methacrylates, being small reactive molecules, can penetrate the skin and bind to proteins, forming hapten–protein complexes that activate T cells and cause allergic contact dermatitis, while irritant dermatitis arises through non-immunologic mechanisms (Azeem et al., 2020).

### 1.3 Cytotoxicity, apoptosis, and necrosis

Beyond their immunological effects, methacrylates can also directly affect cell viability and function. Cytotoxins are agents capable of damaging cells or impairing cellular function and viability, while cytotoxicity refers to the capacity of a substance to induce such adverse cellular effects. Methacrylates are generally considered mildly to moderately cytotoxic (Kostić et al., 2022; Schmalz et al., 2000). However, Nilsen et al. emphasized the need for standardized terminology and methods to interpret *in vitro* findings on resin-based materials since the terminology and interpretation of achieved reactions are difficult (Nilsen et al., 2016). Released methacrylate monomers can exert cytotoxic effects at sufficient concentrations, although their biological activity may vary depending on their chemical structure and physicochemical properties (Yoshii, 1997).

Cell death induced by cytotoxic exposure may occur through distinct mechanisms, including apoptosis, a controlled and generally non-inflammatory form of cell death, or necrosis, an uncontrolled, energy-deprived process leading to membrane rupture and inflammation (Elmore, 2007).

Although previous studies and reviews have examined the biological and cytotoxic effects of dental methacrylates, no narrative review to date has specifically focused on the combined immunomodulatory and cytotoxic effects of the low–molecular-weight methacrylates HEMA and TEGDMA in the context of host immune responses and inflammatory signaling.

## 2 Aim

The aim of this narrative review was to summarize and critically discuss current evidence on the immunomodulatory and cytotoxic effects of the low-molecular-weight dental methacrylates HEMA and TEGDMA. Emphasis was placed on their effects on immune-related outcomes when applied individually, in combination, or in the presence of microbial components (lipopolysaccharide (LPS) and lipoteichoic acid (LTA)), as reported in experimental *in vitro* and *in vivo* studies.

## 3 Materials and methods

### 3.1 Literature search and study selection

A narrative literature review was conducted to summarize and critically discuss experimental evidence on the immunological and cytotoxic effects of dental methacrylates. This review was designed as a narrative review with the aim of providing an overview of current knowledge regarding the immunomodulatory and cytotoxic effects of HEMA and TEGDMA. Given the diversity of the available literature, including differences in experimental models, exposure conditions, concentrations, and outcome measures, a narrative approach was considered more appropriate than a formal systematic review methodology (Sukhera, 2022), and the feasibility of formal systematic comparison and quantitative synthesis was therefore limited.

The literature search was last updated in January 2024. An electronic search was conducted in MEDLINE (PubMed) using combinations of the following keywords: methacrylate; polymer; monomer; 2-hydroxyethyl methacrylate; HEMA; triethylene glycol dimethacrylate; TEGDMA; dental; dentistry; oral; immune system; antibody; immunoglobulin; interleukin; cytotoxicity; lipopolysaccharide; LPS; lipoteichoic acid; and LTA. The search strategy was intended to support the narrative synthesis and improve transparency regarding the literature identification process, rather than to constitute a formal systematic review.

Inclusion criteria comprised full-length original research articles published in English that investigated interactions between dental methacrylates and immune-related or cytotoxic outcomes using *in vitro* or *in vivo* experimental models, with a primary focus on HEMA and/or TEGDMA. Studies published in other languages, unavailable full texts, conference abstracts, and poster presentations were excluded.

In total, 42 publications published between 1997 and 2024 were considered relevant to the scope of the present review and are summarized in Table 1.

**TABLE 1:** Summary of experimental studies on methacrylate compounds, concentrations, and tested cell Types/animals. The table presents a summary of findings from the different studies (n = 42). The table include the specific type of methacrylate studied, the concentrations of methacrylate compounds used, and the cell types/animals tested. Each reference corresponds to a study that examined the effects of different methacrylate concentrations on a particular cell type/animal, highlighting the variability in compound usage and cellular response. Reported concentrations are based on the information provided in the original studies and may refer either to the full experimental concentration range tested or to concentrations associated with significant observed biological effects.

References	Author	Concentration range	Methacrylate	Cells/Animals	Findings
Schmalz et al. (2000)	Schmalz et al.	0–100 mM	TEGDMA	TR146 cells isolated from a squamous cell carcinoma	Incresead IL-6 och IL-8, slightly reduced cell viability even at very high concentrations of 30 mM and 100 mM
Alizadehgharib et al. (2020)	Alizadehgharib et al.	500 μM	TEGDMA	Human neutrophils	Increased IL-8, increased cell death and formation of neutrophil extracellular traps
Alizadehgharib et al. (2018)	Alizadehgharib et al.	0–1000 μM	HEMA	THP-1 cells and human peripheral blood mononuclear cells	Increased IL-1β and IL-18 secretion and NLRP3 formation
Alizadehgharib et al. (2018)	Alizadehgharib et al.	0–20 μmol/mouse	HEMA +/- KCl	Mice	Inflammatory skin responses that is suppressed by including KCl
Alizadehghari et al. (2018)	Alizadehgharib et al.	0–1000 μM and 20μmol/mouse	TEGDMA	Human peripheral blood mononuclear cells and mice	Increased IL-1β, IL-6, IL-8, IL-18, GRO-α and MCP-1 and TNF-α. TNF-α decreased in higher concentration. TEGDMA exerted adjuvant properties in mice
Trubiani et al. (2012)	Trubiani et al.	0–5 mmol L−1	HEMA	Dental pulp mesenchymal stem cells	Increased IL-6 and IL-8 secretion
Alizadehgharib et al. (2017)	Alizadehgharib et al.	0–1000 μM	HEMA or TEGDMA	Human peripheral blood mononuclear cells	Increased IL-1β, IL-8, IL-18, and VEGF secretion
Alizadehgharib et al. (2017)	Alizadehgharib et al.	0–1000 μM	TEGDMA	Human peripheral blood mononuclear cells	Increased IL-6 and TNF-α secretion
Alizadehgharib et al. (2017)	Alizadehgharib et al.	0–20 μmol/mouse	HEMA + OVA	Mice	Increased IgG anti-OVA antibody level
Alizadehgharib et al. (2017)	Alizadehgharib et al.	0–20 μmol/mouse	TEGDMA + OVA	Mice	Increased IgG anti-OVA antibody level
Alizadehgharib et al. (2017)	Alizadehgharib et al.	0–1000 μM	HEMA or TEGDMA	Human peripheral blood mononuclear cells	Slightly reduced cell viability 90%–95%
Noda et al. (2003)	Noda et al.	HEMA, 0–1.2 mmol/L and TEGDMA 0–0.75 mmol/L	HEMA or TEGDMA	THP-1 cells	No TNF-α secretion
Noda et al. (2003)	Noda et al.	HEMA, 0–1.2 mmol/L and TEGDMA 0–0.75 mmol/L	HEMA or TEGDMA	THP-1 monocytes	Reduced LPS-induced TNF-α secretion
Mantellini et al. (2006)	Mantellini et al.	0–1000 nM	HEMA	Mouse odontoblast-like cells and in macrophages	Increased VEGF secretion
Mantellini et al. (2006)	Mantellini et al.	0–1000 nM	HEMA	Undifferentiated pulp cells and fibroblasts	No VEGF secretion
Gregson et al. (2008)	Gregson et al.	0–1.25 mM	TEGDMA	U937 cells	Increased MCP-1 secretion
Moharamzadeh et al. (2008)	Moharamzadeh et al.	-	TEGDMA-based composite resin	Three-dimensional human oral mucosal model	Increased IL-1β secretion
Moharamzadeh et al. (2009)	Moharamzadeh et al.	-	TEGDMA	Tissue-engineered human oral mucosal model	Increased IL-1β secretion
Andersson and Dahlgren (2010)	Andersson et al.	0–750 μmol/L	HEMA	B-lymphocytes	Increased IgG1, not in IgM or IgA
Sandberg et al. (2005)	Sandberg et al.	0–20 μmol/mouse	HEMA + OVA	Mice	Increased levels of IgG and IgE anti-OVA antibodies
Sandberg et al. (2005)	Sandberg et al.	0–20 μmol/mouse	HEMA	Mice	Inflammatory skin responses
Andersson and Dahlgren (2011a)	Andersson et al.	0–20 μmol/mouse	HEMA + OVA	Mice	Increased anti-OVA IgG activity, no anti-OVA IgM activity,increased baseline and concanavalin A-stimulated (ConA) in vitro proliferation of splenocytes
Sandberg et al. (2002)	Sandberg et al.	23 μL	HEMA-conjugated mouse serum albumin (H-MSA)	Mice	Increased production of IgG antibodies to native MSA
Andersson and Dahlgren (2011b)	Andersson et al.	0.161 μg or 3.6 ng of HEMA h−1	Osmotic pumps delivering HEMA + OVA	Mice	No difference in anti-OVA IgG or IgM activity, no signs of inflammation in biopsies, decreased production of anti-OVA IgA as well as affected the growth of mice, decreased ConA-stimulated in vitro proliferation of splenocytes
Östberg et al. (2018)	Östberg et al.	0–20 μmol/mouse	HEMA + OVA/S. mutans	Mice	Adjuvant properties
Bando et al. (2014)	Bando et al.	1% and 20 µL/ear	HEMA	Mice	Adjuvant properties + increased IL-1α and IL-1β secretion
Bølling et al. (2019)	Bolling et al.	0–200 μM	HEMA or TEGDMA, or HEMA and TEGDMA in combination	RAW264.7 macrophages	Reduced the LPS-induced IL-1β alone and in combination, and in combination also reduced the LPS-induced TNF-a secretion. No interference with LPS-induced pre-transcriptional signaling pathways
Bølling et al. (2019)	Bolling et al.	0–200 μM	HEMA or TEGDMA, or HEMA and TEGDMA in combination	RAW264.7 macrophages	No reduced cell viability as compared to control cells
Bølling et al. (2013)	Bolling et al.	0.5–2 mM	HEMA	THP-1 monocytes macrophages	Reduced S.aureus-induced cytokine release
Eckhardt et al. (2009)	Eckhardt et al.	0–2 mM	TEGDMA	RAW264.7 macrophages	Reduced LPS-induced TNF-α, IL-6, and IL-10 secretion, supressed expression of CD14, CD40, CD80, CD86, and MHC class I
Krifka et al. (2010)	Krifka et al.	0–3 mM	TEGDMA	RAW264.7 macrophages	Reduced LPS-induced TNF-α, IL-6 and IL-10 secretion
Rakich et al. (1999)	Rakich et al.	0–4000 mol/L	HEMA	THP-1 cells	Suppressed LPS-induced IL-1β and TNF-α secretion
Schweikl et al. (2018)	Schweikl et al.	0–8 mM	HEMA	RAW 264.7 macrophages	Attenuation of LPS-stimulated NF-kB activation
Schweikl et al. (2018)	Schweikl et al.	0–4 mM	HEMA	RAW 264.7 macrophages	Slightly reduced the percentage of viable cells (95%–93%)
Schweikl et al. (2022)	Schweikl et al.	0–8 mM	HEMA	Human pulp fibroblasts and primary cells from the dentin-pulp interface	Reduced LPS- and LTA-induced IL-6 secretion
Neves et al. (2019)	Neves et al.	0–2150.0 μM	TEGDMA	peripheral blood mononuclear cells	Redcued the P. gingivalis-induced IL-1β and TNF-α secretion
Neves et al. (2019)	Neves et al.	0–10 mM	TEGDMA	Human peripheral blood mononuclear cells	Induced dose-dependent cytotoxicity
Mathisen et al. (2015)	Mathisen et al.	0–50 μM	TEGDMA	RAW 264.7 macrophages	Reduced LPS-induced IL-1β secretion
Andersson and Dahlgren (2008)	Andersson et al.	0–15 mM	HEMA	Neutrophils	Reduced neutrophil’s ability to kill ingested bacteria
Ginzkey et al. (2015)	Ginzkey et al.	HEMA 0μM-1 mM and TEGDMA 0μM–100 μM	HEMA or TEGDMA	Human lymphocytes	Genotoxic effects and dose-dependent increase in the frequence of chromosome aberrations and sister chromatid exchange
Schweikl et al. (2006)	Schweikl et al.	-	HEMA and TEGDMA	-	Review of the genetic and cellular toxicology of dental resin monomers
Nilsen et al. (2018)	Nilsen et al.	0–5 mM	TEGDMA	THP-1 cells	Expressed protein expression profile associated with oxidative stress, DNA damage, mitochondrial dysfunction, cell cycle inhibition and altered immune genes
Fukumoto et al. (2013)	Fukumoto et al.	0–9.6 mM	HEMA	THP-1 cells	Induced significant expression of both CD86 and CD54
Krifka et al. (2011)	Krifka et al.	0–3 mM	TEGDMA	RAW264.7 macrophages and HeLa cell cultures	Induced DNA damage and initiated downstream of MAP kinases
Samuelsen et al. (2007)	Samuelsen et al.	HEMA 0–15 mM and TEGDMA 0–4 mM	HEMA or TEGDMA	Rat submandibular salivary gland acinar cells	Induced ROS formation and differential MAP kinase activation that is involved in the apoptotic response caused by the monomers
Ansteinsson et al. (2011)	Ansteinsson et al.	0–5.4 mM	HEMA	Lung epithelial cell line BEAS-2B	Induced DNA-damage, depletion of GSH amd increased levels of ROS
Morisbak et al. (2015)	Morisbak et al.	0–10 mM	HEMA	Lung epithelial cell line BEAS-2B	Reduced the cell viability, caused depletion of GSH amd increased levels of ROS
Samuelsen et al. (2019)	Samuelsen et al.	0–8 mM	HEMA	THP-1 cells	Reduced cell viability, depletion of GSH and increased levels of ROS
Schweikl et al. (2016)	Schweikl et al.	0–8 mM	HEMA	RAW264.7 macrophages	Reduced cell viability. LPS-induced apoptosis was neutralized by 4–6 mM HEMA
Paranjpe et al. (2005)	Paranjpe et al.	0–0.082 M	HEMA	Human peripheral blood mononuclear cells from healthy donors and HEMA-sensitized patients and RAW 264.7 macrophages	Induced apoptosis in a dose-dependent manner
Harorli et al. (2009)	Harorli et al.	0–8 mM	TEGDMA	THP-1 cells	Increasing TEGDMA concentrations caused a decrease in cell viability, cell proliferation was inhibited
Heil et al. (2002)	Heil et al.	HEMA 300 000 umol L−1 TEGDMA 10 000 umol L−1	HEMA or TEGDMA	THP-1 cells and Human peripheral blood mononuclear cells	THP-1 cells were more sensitive regarding cytotoxicity the monomers, and HEMA and TEGDMA supressed the LPS-induced TNF-α secretion
Becher et al. (2006)	Becher et al.	0–2000 μg/mL	HEMA or TEGDMA	Alveolar macrophages (rats) and J774A1 mouse macrophages	TEGDMA is more cytotoxic than HEMA while HEMA caused had a higher increase in numbers of apoptotic cells compared to TEGDMA.

Abbreviation: BEAS, Bronchial epithelial cells; CD14, Cluster of differentiation 14; CD40, Cluster of differentiation 40; CD54, Cluster of differentiation 54; CD80, Cluster of differentiation 80; CD86, Cluster of differentiation 86; DNA, Deoxyribonucleic acid; GRO, Growth-regulated oncogene; GSH, Glutathione; HEMA, 2-Hydroxyethyl methacrylate; IL, Interleukin; LPS, Lipopolysaccharide; LTA, Lipoteichoic acid; MAP, Mitogen-activated protein; MCP, Monocyte chemoattractant protein; MHC, Major histocompatibility complex; MSA, Methacrylic acid; NF-κB, Nuclear factor kappa-light-chain-enhancer of activated B cells; NLRP3, NOD-like receptor family pyrin domain containing 3; OVA, Ovalbumin; RAW, Murine macrophage cell line; ROS, Reactive oxygen species; TEGDMA, Triethylene glycol dimethacrylate; THP, Human monocytic leukemia cell line (THP-1); TNF, Tumor necrosis factor; TR146, Human buccal carcinoma cell line; VEGF, Vascular endothelial growth factor.

### 3.2 Use of AI

During the preparation of this manuscript, generative artificial intelligence (ChatGPT, OpenAI, San Francisco, CA, USA) was used to assist with language editing and improvement of readability. The authors reviewed and edited all generated content and take full responsibility for the accuracy and integrity of the manuscript.

## 4 Results and discussion

### 4.1 *In vitro* immunomodulation

Resin monomers have been widely investigated for their potential to induce inflammatory responses. Several studies have reported increased cytokine secretion after exposure to HEMA and/or TEGDMA (Alizadehgharib et al., 2020; Alizadehgharib et al., 2018; Alizadehghari et al., 2018). In a 3D epithelial model, TEGDMA induced a dose-dependent rise in IL-6 and IL-8, with IL-6 peaking at 30 mM before declining at higher, non-cytotoxic concentrations (Schmalz et al., 2000). However, the concentrations applied in this study were substantially higher than those used in several other experimental models, and the limited cytotoxic effects reported contrast with findings from multiple monolayer-based cell culture studies. These differences may partly reflect the use of a 3D epithelial model, which may more closely resemble tissue architecture and diffusion conditions *in vivo*, thereby influencing cellular sensitivity and cytokine responses. Consequently, direct comparison between this model and conventional monolayer cultures should be interpreted with caution. Similarly, HEMA increased IL-6 and IL-8 production in dental pulp mesenchymal stem cells *in vitro* (Trubiani et al., 2012), both cytokines being key mediators of inflammation (Al-Qahtani et al., 2024). In PBMCs, 500 μM HEMA or TEGDMA elevated IL-1β, IL-8, and IL-18, with TEGDMA also enhancing IL-6 and TNF-α. At 1000 μM, cytokine release was reduced, suggesting a non-linear dose–response relationship, potentially reflecting cytotoxic or stress-related effects at higher concentrations (Alizadehgharib et al., 2017). However, THP-1 monocytes did not secrete TNF-α after exposure to either monomer (Noda et al., 2003). Notably, these divergent findings across cell types indicate that cytokine responses to methacrylates are highly model-dependent, which complicates direct comparison between studies and limits the generalizability of individual findings. Collectively, these findings indicate that methacrylate exposure can induce early cytokine responses consistent with activation of innate immune pathways. At higher concentrations, the observed reduction in cytokine release may reflect a shift toward cellular stress or cytotoxic effects rather than sustained inflammatory signaling. This concentration-dependent pattern may contribute to inconsistencies across studies.

Beyond classical inflammatory mediators, HEMA (1000 nM) was also shown to induce VEGF release in odontoblast-like cells and macrophages, but not in pulp cells or fibroblasts, suggesting a possible involvement in pulp neovascularization following adhesive resin application (Mantellini et al., 2006). Furthermore, exposure to HEMA or TEGDMA has also been associated with increased IL-1β levels and MCP-1 production *in vitro* (Gregson et al., 2008; Moharamzadeh et al., 2008; Moharamzadeh et al., 2009). Supporting a mechanistic basis for these inflammatory responses, exposure of PBMCs and THP-1 cells to HEMA (1000 μM) was shown to activate the NLRP3 inflammasome, resulting in increased secretion of IL-1β and IL-18. Blocking NLRP3 activation with KCl attenuated this response, confirming inflammasome involvement in HEMA-induced cytokine release (Alizadehgharib et al., 2018). These mechanistic findings strengthen the biological plausibility of methacrylate-induced inflammation, although most evidence is derived from controlled *in vitro* conditions that may not fully reflect the complexity of tissue-level responses *in vivo*.

In addition to the altered cytokine responses, Andersson et al. (Andersson and Dahlgren, 2010) reported that human B cells exposed to low concentrations of HEMA (15 μM and 37 μM) showed a significant increase in IgG1, but not IgM, production, whereas higher concentrations (750 μM) suppressed both. IgA levels remained unchanged compared with unexposed controls. This concentration-dependent effect on antibody production suggests that HEMA may exert both immunostimulatory and immunosuppressive effects depending on exposure conditions.

Taken together, *in vitro* studies indicate that HEMA and TEGDMA can modulate cytokine and immunoglobulin expression in a concentration-, cell type–, and exposure-dependent manner. While several studies report pro-inflammatory effects, others show limited or absent cytokine induction, likely reflecting differences in experimental models, including cell types, exposure duration, and concentration ranges. Most research in this field is based on *in vitro* models, which allow detailed mechanistic insight under controlled conditions but do not fully capture the complexity of *in vivo* immune responses. Consequently, the effects observed *in vivo* may differ, as methacrylates can undergo degradation or metabolism into potentially more immunogenic compounds (Santerre et al., 2001). Moreover, the concentrations used *in vitro* sometimes exceed those expected under clinical conditions, which may further limit the direct translational relevance of these findings. Clinically, these findings underscore the importance of adequate polymerization, as incomplete curing of HEMA or TEGDMA may increase the risk of local inflammatory responses and potentially affect pulp vitality.

### 4.2 *In vivo* immunomodulation

Previous studies have examined the inflammatory and adjuvant effects of HEMA following subcutaneous injection with a protein antigen. Ovalbumin (OVA) is commonly used as a reporter antigen to assess adjuvant activity. Mice immunized with OVA and HEMA show higher serum levels of anti-OVA IgG and IgE compared to those given OVA alone (Alizadehgharib et al., 2017; Sandberg et al., 2005; Andersson and Dahlgren, 2011a). The presence of HEMA increased anti-OVA IgG levels 5.7-fold, while no significant change was observed in anti-OVA IgM activity (Andersson and Dahlgren, 2011a). Similarly, HEMA-conjugated mouse serum albumin (MSA) effectively induced IgG antibodies against native MSA (Sandberg et al., 2002). In another study, mice implanted with osmotic pumps releasing HEMA or NaCl for 40 days and immunized with OVA showed significantly lower anti-OVA IgA levels in the HEMA group, with no difference in IgG or IgM activity (Andersson and Dahlgren, 2011b). Beyond subcutaneous injection, HEMA monomers applied to the sublingual mucosa of BALB/c mice also exhibited adjuvant activity when co-administered with OVA or live *Streptococcus mutans* (Östberg et al., 2018). These adjuvant effects are thought to involve macrophages and IL-1 (Bando et al., 2014). More limited evidence suggests similar adjuvant effects for other methacrylates, as mice receiving OVA with TEGDMA developed higher anti-OVA IgG levels than controls (Alizadehgharib et al., 2017). Collectively, these findings support the notion that HEMA can function as an immune adjuvant *in vivo*, although the magnitude and nature of this effect vary depending on experimental design.

Mice exposed to subcutaneous HEMA injections in the tail exhibited clear inflammatory skin responses (Alizadehgharib et al., 2018; Sandberg et al., 2005), whereas biopsies near osmotic pump openings after 40 days of HEMA administration showed no inflammation (Andersson and Dahlgren, 2011b). Despite the absence of overt local inflammation, long-term HEMA exposure via osmotic pumps affected mouse growth (Andersson and Dahlgren, 2011b). These divergent findings suggest that the inflammatory outcome of HEMA exposure *in vivo* depends on exposure route, dose, and duration.

As previously mentioned, localized inflammation has been observed in the tails of mice subcutaneously injected with HEMA (Alizadehgharib et al., 2018). This local inflammation has been suggested to result from the activation of the NLRP3 inflammasome and could be suppressed when KCl was included in the injection (Alizadehgharib et al., 2018). KCl likely inhibits NLRP3 activation by preserving intracellular potassium levels, which are critical for preventing inflammasome assembly. To date, no *in vivo* animal studies have specifically examined inflammatory skin responses following exposure to TEGDMA. This represents a notable gap in the literature, particularly given the widespread clinical use of TEGDMA and its demonstrated immunomodulatory effects *in vitro*. Consequently, much of the current understanding regarding the immunomodulatory and cytotoxic effects of TEGDMA is derived from *in vitro* experimental models. Although several studies indicate that TEGDMA may exert greater cytotoxic potential than HEMA under certain experimental conditions, caution is warranted when relating these findings directly to clinical *in vivo* situations, where exposure concentrations, tissue barriers, metabolic processes, and local environmental factors may substantially influence biological responses.

In addition to the adjuvant and inflammatogenic properties of HEMA, repeated subcutaneous exposure to HEMA *in vivo* (twice with 3 weeks between exposures) has been shown to increase both baseline and concanavalin A (ConA)-stimulated *in vitro* proliferation of splenocytes (Andersson and Dahlgren, 2011a). However, long-term exposure to low doses of HEMA delivered via osmotic pumps resulted in decreased ConA-stimulated splenocyte proliferation (Andersson and Dahlgren, 2011b), indicating that prolonged exposure may shift immune responses toward immunosuppression rather than sustained activation. This bidirectional effect suggests that short-term exposure may favor immune activation, whereas prolonged exposure may lead to immune suppression.

Overall, the *in vivo* evidence indicates that HEMA, and to a lesser extent TEGDMA, exert adjuvant-like effects and modulate immune responses in a context-dependent manner. As both HEMA and TEGDMA can enhance immune activity, they may contribute to allergic reactions or sensitization to other antigens, such as oral bacteria, food components, or latex proteins. Allergic contact dermatitis induced by these monomers has been reported (Wrangsjö et al., 2001; Kiec-Świerczyńska, 1996). It can be speculated that their adjuvant properties may contribute to this response, however, the precise mechanisms linking adjuvant activity to clinical allergy remain incompletely understood. In particular, the relationship between experimental adjuvant effects observed in animal models and clinically observed hypersensitivity reactions remains insufficiently characterized and warrants further investigation.

Clinically, these findings suggest that exposure to uncured or residual monomers may promote local inflammation and immune activation, particularly in susceptible individuals. This underscores the importance of careful material selection and handling, particularly for individuals with known hypersensitivity or occupational methacrylate exposure. Nevertheless, well-designed human studies are lacking, and the extent to which these experimental findings translate into clinically significant immune modulation in patients remains unclear.

### 4.3 Co-exposure effects

Recent research has examined reactions resulting from co-exposure to HEMA/TEGDMA and bacterial components. Multiple studies have explored how these monomers interact with stressors such as LPS or LTA from oral microorganisms (Noda et al., 2003; Bølling et al., 2019; Bølling et al., 2013; Eckhardt et al., 2009; Krifka et al., 2010; Rakich et al., 1999; Schweikl et al., 2018; Schweikl et al., 2022; Neves et al., 2019). HEMA suppresses LPS- and LTA-induced IL-6 release from human pulp fibroblasts and dentin–pulp interface cells (Schweikl et al., 2022), while TEGDMA strongly downregulates LPS-induced TNF-α, IL-6, and IL-10 expression in RAW264.7 macrophages (Eckhardt et al., 2009; Krifka et al., 2010). Neither compound alone induces TNF-α secretion in THP-1 cells, and both inhibit LPS-induced TNF-α and IL-1β (HEMA) secretion (Noda et al., 2003; Rakich et al., 1999). Similarly, HEMA and TEGDMA, alone or combined, reduce LPS-induced IL-1β release from murine RAW264.7 macrophages (Bølling et al., 2013; Mathisen et al., 2015). The suppression of LPS-induced cytokine secretion by HEMA is attributed to attenuation of NF-κB activation, potentially linked to increased intracellular hydrogen peroxide and nitric oxide levels (Schweikl et al., 2018). In addition to these effects, HEMA has also been shown to reduce *Staphylococcus aureus (S. aureus*)–induced cytokine release from macrophages pre-exposed to HEMA and subsequently challenged with *S. aureus* (Bølling et al., 2019). Similarly, co-exposure to TEGDMA and heat-inactivated *Porphyromonas gingivalis* for 5 h led to reduced bacteria-induced release of IL-1β and TNF-α from human PBMCs (Neves et al., 2019). Collectively, these findings indicate that HEMA and TEGDMA can suppress microbe-induced inflammatory signaling, further underscoring the context-dependent nature of their immunomodulatory effects. Importantly, this suppressive effect contrasts with the pro-inflammatory responses observed under isolated monomer exposure, highlighting their dual immunomodulatory profile.

The observed attenuation of microbe-induced cytokine responses following HEMA and TEGDMA co-exposure may involve interactions between oxidative stress pathways and redox-sensitive inflammatory signalling. NF-κB activity is influenced by intracellular ROS levels, suggesting that monomer-induced oxidative stress may contribute to dysregulated inflammatory signaling under certain experimental conditions. Furthermore, reduced cytokine production in co-exposure models may reflect both cytotoxic effects impairing cellular viability and more direct immunomodulatory effects on inflammatory signaling pathways. This distinction is important when interpreting the potential clinical relevance of these findings.

Furthermore, RAW264.7 macrophages exposed to 25 μg/mL LPS (*Escherichia coli*) show increased expression of CD14, CD40, CD80, CD86, and MHC I, whereas co-exposure to TEGDMA reduces or inhibits the expression of these antigens (Eckhardt et al., 2009). This suggests that TEGDMA may impair antigen presentation and co-stimulatory signaling, thereby limiting an immune cell’s ability to initiate an effective response to pathogens and their byproducts. In contrast, 24-h LPS treatment increased CD14 expression, and co-exposure to TEGDMA or HEMA did not alter this effect (Bølling et al., 2013). Beyond modulating bacteria-induced immune responses, HEMA has also been shown to reduce neutrophil bactericidal activity (Andersson and Dahlgren, 2008). Together, these findings indicate that methacrylates may interfere not only with cytokine signaling but also with key cellular functions required for effective innate immune defense.

Overall, co-exposure to HEMA and TEGDMA interferes with immune responses induced by bacterial components such as LPS and LTA. TEGDMA decreases LPS-induced TNF-α, IL-1β, IL-6, and IL-10, whereas HEMA reduces TNF-α, IL-1β, and IL-6 expression (Noda et al., 2003; Bølling et al., 2013; Eckhardt et al., 2009; Schweikl et al., 2022). LTA and LPS are immunogenic pathogen-associated molecular patterns (PAMPs) that bind to pattern recognition receptors, such as Toll-like receptors, and activate innate immune signaling pathways, leading to the release of pro-inflammatory (e.g., IL-1, IL-6, TNF-α) and anti-inflammatory cytokines (e.g., IL-10) (Galler et al., 2021). As key effectors of the innate immune system, macrophages coordinate inflammatory responses and play an essential role in tissue repair. By attenuating cytokine release and immune cell activation, HEMA and TEGDMA may compromise host defense against oral bacteria. In the context of chronic inflammatory conditions such as periodontitis, this immunosuppressive effect may have important implications. While reduced inflammatory signaling could theoretically limit tissue damage associated with chronic inflammation, suppression of macrophage activation and cytokine production may also impair bacterial clearance and host defense mechanisms. Since periodontal disease involves interactions between oral biofilms and host immune responses, altered immune reactivity caused by HEMA or TEGDMA exposure could potentially influence both inflammation and bacterial survival. However, the clinical relevance of these experimental observations remains uncertain and requires further investigation under clinically relevant exposure conditions.

Since macrophages are involved in the healing of tissues, they may also influence the regeneration of injured dental pulp and dentin (Kim et al., 2019). Consequently, if immune responses are attenuated, it may affect pulp healing and suppress the establishment of a resistant and impermeable barrier against pathogen internalization. Reduced production of inflammatory cytokines may also impair bacterial clearance, which may promote biofilm formation and contribute to caries development.

However, the balance between protective anti-inflammatory effects and detrimental suppression of host defense remains poorly defined and is likely to depend on the local microenvironment and exposure conditions. In particular, factors such as bacterial load, biofilm composition, and local tissue status may critically influence whether these effects are beneficial or harmful. Additionally, most available *in vivo* studies rely on animal models using exposure conditions that may not directly reflect clinical scenarios in dentistry, thereby limiting their translational relevance.

### 4.4 Cytotoxic mechanisms

Beyond their immunomodulatory effects, the cytotoxic and genotoxic properties of HEMA and TEGDMA are of particular concern due to their potential impact on host tissues. These monomers can interact with proteins, lipids, and DNA, posing risks of genotoxicity and cytotoxicity (Sandberg et al., 2002; Ginzkey et al., 2015; Schweikl et al., 2006; Nilsen et al., 2018). In addition to direct DNA damage, previous studies have suggested that methacrylate-induced oxidative stress may also contribute to mutagenic effects, further emphasizing the potential biological consequences of prolonged or repeated exposure to dental monomers (Schweikl and Schmalz, 1999).

The sensitization potential of HEMA, demonstrated by the increased expression of costimulatory factors CD54 and CD86 in THP-1 cells, has been linked to oxidative stress, which is closely associated with the production of reactive oxygen species (ROS). This suggests that redox imbalance may contribute both to cytotoxicity and immune activation (Fukumoto et al., 2013). ROS production has also been identified as an important contributor to resin monomer–induced cellular responses, including altered innate immunity, impaired dentin mineralization, genotoxicity, and delayed cell cycle progression (Krifka et al., 2011). Together, these findings indicate that oxidative stress represents a unifying mechanism linking cytotoxic and immunomodulatory effects of methacrylates.

Krifka et al. demonstrated that TEGDMA disrupts cellular regulatory pathways by affecting transcription factors activated either in response to DNA damage or via signaling cascades downstream of mitogen-activated protein (MAP) kinases (Krifka et al., 2011). Additionally, apoptotic responses triggered by exposure to TEGDMA and HEMA appear to be linked to ROS formation and differential activation of MAP kinases (Samuelsen et al., 2007). These mechanistic insights support the biological plausibility of the observed cellular effects, although their relative contribution under clinically relevant exposure conditions remains unclear.

Moreover, HEMA exhibits cytotoxic effects in several cell types, including human lung epithelial cells, where its potential to induce DNA damage has been suggested as a contributing factor (Samuelsen et al., 2007). Exposure to HEMA leads to cellular glutathione (GSH) depletion and increased ROS levels (Ansteinsson et al., 2011), findings consistent with previous observations in human lung epithelial cells (Morisbak et al., 2015). Since GSH represents one of the major intracellular antioxidant defense systems, its depletion may disrupt redox homeostasis and contribute to intracellular ROS accumulation, oxidative stress, and subsequent cellular damage. Furthermore, exposure to HEMA (1–8 mM) for 24 h reduced cell viability in THP-1 cells while increasing the expression of several cytoprotective proteins, consistent with an elevated oxidative burden caused by GSH depletion and the electrophilic nature of HEMA (Samuelsen et al., 2019). These adaptive cellular responses likely represent protective mechanisms aimed at restoring redox balance under conditions of oxidative stress. However, persistent or excessive activation of such stress-response pathways may also indicate sustained oxidative stress that, if unresolved, may contribute to subsequent cellular dysfunction, apoptosis, or necrosis.

While several studies have specifically investigated apoptotic signaling pathways following methacrylate exposure, other studies have primarily assessed broader cytotoxic outcomes such as reduced metabolic activity or impaired cell viability. These endpoints are related but measure different biological processes, and differences in experimental methodology should therefore be considered when comparing results across studies.

Apart from mechanistic studies, several studies have also explored the effects of HEMA and TEGDMA on cell viability and apoptosis. Cells exposed to 500–1000 μM HEMA or TEGDMA for 24 h maintained relatively high viability (90%–95%), whereas exposure to the acrylate monomer DEGDA at the same concentrations resulted in markedly reduced viability (<50%) (Alizadehgharib et al., 2017). Schmalz et al. reported that even at concentrations as high as 30 mM and 100 mM, TEGDMA caused only minor reductions in cell viability in tissue culture models (Schmalz et al., 2000). However, these findings contrast with studies demonstrating dose-dependent cytotoxicity of TEGDMA in PBMCs, highlighting variability between experimental models and cell types (Neves et al., 2019).

Similarly, exposure to 1–4 mM HEMA had minimal effects on RAW264.7 macrophage viability (93%–95%), whereas 8 mM HEMA significantly reduced viability to 53% (Schweikl et al., 2018). Interestingly, co-treatment with 0.1 μg/mL LPS improved cell viability; in the presence of 4 mM HEMA, viability increased from 57% to 72%, and at 8 mM HEMA, viability improved from 53% to 82% (Schweikl et al., 2018). These findings are supported by additional studies showing enhanced viability of RAW264.7 macrophages exposed to HEMA in the presence of LPS, particularly at higher concentrations (Schweikl et al., 2016). This interaction between methacrylates and microbial components suggests that cytotoxic effects may be modulated by the inflammatory microenvironment, further complicating the interpretation of isolated *in vitro* findings.

Bølling et al. reported that exposure of RAW264.7 macrophages to 5–200 μM TEGDMA or HEMA, alone or in combination, did not affect cell viability compared with controls (Bølling et al., 2013). In contrast, other studies demonstrated that HEMA induces dose-dependent apoptosis in PBMCs from healthy donors, HEMA-sensitized patients, and RAW264.7 macrophages (Paranjpe et al., 2005). Similarly, increasing concentrations of TEGDMA reduced THP-1 cell viability through apoptosis (Harorli et al., 2009). These findings suggest pronounced cell-type–dependent differences in sensitivity to methacrylate-induced cytotoxicity, with THP-1 cells reported to be 3–25 times more sensitive to HEMA- and TEGDMA-induced cytotoxicity and TNF-α responses than PBMCs (Heil et al., 2002).

Interpretation of cytotoxic effects across studies should be approached with caution, as different experimental endpoints may reflect different biological processes. Reduced cell viability observed in metabolic activity assays does not necessarily correspond directly to apoptosis and may also reflect necrosis, impaired proliferation, or altered cellular metabolism. Therefore, direct comparison between studies using different analytical approaches, including viability assays and apoptosis-specific methods, is difficult and may contribute to variability in reported effective concentrations.

Taken together, *in vitro* evidence demonstrates that HEMA and TEGDMA can induce oxidative stress, disrupt redox homeostasis, and trigger apoptosis in a concentration- and cell type–dependent manner (Ginzkey et al., 2015; Schweikl et al., 2006; Nilsen et al., 2018; Samuelsen et al., 2019). Overall, the findings suggest that both monomers possess cytotoxic potential, with TEGDMA appearing to be slightly more cytotoxic than HEMA (Heil et al., 2002; Becher et al., 2006; Geurtsen et al., 1998). Although the precise mechanisms underlying this difference remain incompletely understood, it may partly relate to structural and physicochemical differences between the monomers. TEGDMA contains two methacrylate functional groups, whereas HEMA contains only one, which may increase its chemical reactivity and capacity to interfere with intracellular signaling pathways and redox homeostasis. However, it should be noted that many studies employ concentrations that may exceed those encountered under clinical conditions, which may overestimate their cytotoxic potential. From a clinical perspective, these observations reinforce the importance of adequate polymerization and careful material selection to minimize exposure to residual monomers. Furthermore, the lack of well-defined exposure thresholds for cytotoxic effects represents a key limitation in relating these findings to clinical risk assessment. A detailed understanding of these cytotoxic mechanisms is therefore essential for improving the biocompatibility of resin-based dental materials and reducing the risk of adverse biological reactions.

### 4.5 Clinical implications

The present study compiles current knowledge on the effects of the dental monomers HEMA and TEGDMA on the immune system, with a particular focus on cytokine and antibody expression, as well as their cytotoxic properties. These findings indicate that methacrylates can induce a range of immunomodulatory effects both *in vitro* and *in vivo*, including increased secretion of IL-1β, IL-6, IL-8, TNF-α, and IL-18, along with oxidative stress, DNA damage, and reduced cell viability. Both monomers also exhibit adjuvant-like properties when co-administered with antigens, further highlighting their potential to influence immune responses. Taken together, these findings indicate that HEMA and TEGDMA are biologically active compounds capable of modulating both immune function and cellular viability, rather than being inert components of dental materials.

An important observation emerging from the reviewed literature is the dual nature of the immunological effects of HEMA and TEGDMA. Depending on concentration, exposure duration, and experimental context, these monomers can both stimulate and attenuate immune responses. This bidirectional behavior represents a central challenge in assessing their biological impact, as both excessive immune activation and immune suppression may have clinically relevant consequences. Such variability may partly explain the inconsistent outcomes reported across studies.

An important limitation within the current literature is the discrepancy between experimentally applied monomer concentrations and those likely encountered clinically *in vivo*. Previous analyses have shown that the release of resin monomers from dental materials is generally low under clinical conditions and influenced by factors such as polymerization efficiency, material composition, degradation, and exposure time (Van Landuyt et al., 2011). Nevertheless, several *in vitro* studies have used HEMA and TEGDMA concentrations exceeding those likely encountered in the pulp-dentin complex, oral mucosa, or systemic circulation following placement of resin-based dental materials. While such concentrations may be useful for mechanistic investigations, their clinical relevance remains uncertain. Therefore, caution is needed when relating *in vitro* findings directly to clinical conditions. However, locally increased monomer concentrations near incompletely polymerized materials may still occur and could contribute to local tissue reactions, particularly in deep restorations or compromised tissue barriers.

From a clinical perspective, these findings are relevant, as exposure to uncured or residual HEMA or TEGDMA may occur during restorative procedures. Practitioners should be aware of the risk of adverse reactions when these monomers come into contact with soft tissue or the dental pulp. Such reactions have been described in the literature (Hensten-Pettersen, 1998; Lygre et al., 2003; Mittermüller et al., 2018), underscoring the importance of proper material handling, adequate polymerization, and adherence to recommended curing protocols. In particular, inadequate curing, improper handling, or repeated occupational exposure may increase the likelihood of biologically significant monomer release. However, most available evidence is derived from *in vitro* or animal models, which may not fully reflect clinical exposure scenarios in terms of concentration, exposure time, and tissue complexity. Consequently, caution is warranted when extrapolating experimental findings directly to clinical practice. This gap highlights the need for clinically relevant exposure models that better approximate real-world conditions in the oral environment. Further *in vivo* studies are therefore needed to clarify dose–response relationships, long-term effects, and the relevance of combined exposures to dental monomers and microbial components. In particular, studies integrating material exposure with microbial biofilms and host tissue responses would provide more clinically meaningful insights.

By summarizing and integrating current evidence, this review contributes to a more comprehensive understanding of the immunomodulatory and cytotoxic effects of HEMA and TEGDMA. This knowledge may support the development of resin-based dental materials with improved biocompatibility and reduced immunotoxicity. Understanding the biological impact of these commonly used monomers appears essential not only for patient safety but also for guiding future innovations in restorative dentistry. Moreover, improved understanding of these mechanisms may inform both material design and clinical protocols aimed at minimizing adverse biological effects.

As this work is a narrative review, it is subject to limitations such as potential selection bias and reduced reproducibility compared with systematic reviews. A risk of selection bias cannot be completely excluded, particularly in emerging research areas with a limited number of contributing research groups and heterogeneous experimental methodologies. However, the narrative format allows for broader integration of heterogeneous findings and facilitates a nuanced discussion of complex immunological and cytotoxic mechanisms. Nevertheless, future systematic and quantitative analyses would be valuable to validate and refine the conclusions drawn from the current body of evidence.

## 5 Conclusion

Within the scope of this narrative review, HEMA and TEGDMA are shown to exert both immunosuppressive and immunostimulatory effects, influencing cytokine and immunoglobulin production in a manner dependent on concentration, cell type, and exposure conditions. Their adjuvant-like properties further suggest an ability to modulate immune sensitization, thereby raising concerns regarding the potential development of allergic reactions. Evidence also indicates that co-exposure to HEMA and TEGDMA with bacterial components such as LPS and LTA may attenuate immune responses, potentially affecting host defense mechanisms.

In addition to their immunomodulatory effects, both monomers exhibited cytotoxic potential, including the induction of oxidative stress, DNA damage, and apoptotic responses, with TEGDMA appearing to be slightly more cytotoxic than HEMA. Taken together, these findings highlight the complex and context-dependent biological effects of low–molecular-weight dental methacrylates.

From a clinical perspective, these observations underscore the importance of optimal polymerization protocols and careful material selection, particularly in clinical situations involving proximity to the dental pulp or oral mucosa. Minimizing exposure to residual monomers is essential to reduce the risk of adverse biological reactions and ensure the biocompatibility of resin-based dental materials.

Future studies should prioritize the use of clinically relevant monomer concentrations and standardized experimental exposure conditions to improve the clinical relevance of *in vitro* findings. In addition, further *in vivo* investigations are needed to better clarify the clinical significance of the immunomodulatory effects associated with HEMA and TEGDMA exposure.
